# IL6 sensitizes prostate cancer to the antiproliferative effect of IFNα_2_ through IRF9

**DOI:** 10.1530/ERC-13-0222

**Published:** 2013-08

**Authors:** Holger H H Erb, Regina V Langlechner, Patrizia L Moser, Florian Handle, Tineke Casneuf, Karin Verstraeten, Bettina Schlick, Georg Schäfer, Brett Hall, Kate Sasser, Zoran Culig, Frédéric R Santer

**Affiliations:** 1 Division of Experimental Urology, Department of UrologyInnsbruck Medical University6020, InnsbruckAustria; 2 Department of PathologyInnsbruck Medical University6020, InnsbruckAustria; 3 Oncology Biomarkers, Janssen Research and DevelopmentBeerseBelgium; 4 Oncotyrol Center for Personalized Medicine6020, InnsbruckAustria; 5 Oncology Biomarkers Janssen Research and DevelopmentSpring House, PennsylvaniaUSA

**Keywords:** IRF9, IL6, prostate cancer, IFNα_2_, inflammation

## Abstract

Development and progression of prostate cancer (PCa) are associated with chronic inflammation. The cytokine interleukin 6 (IL6) can influence progression, differentiation, survival, and angiogenesis of PCa. To identify novel pathways that are triggered by IL6, we performed a gene expression profiling of two PCa cell lines, LNCaP and MDA PCa 2b, treated with 5 ng/ml IL6. Interferon (IFN) regulatory factor 9 (IRF9) was identified as one of the most prevalent IL6-regulated genes in both cell lines. IRF9 is a mediator of type I IFN signaling and acts together with STAT1 and 2 to activate transcription of IFN-responsive genes. The IL6 regulation of IRF9 was confirmed at mRNA and protein levels by quantitative real-time PCR and western blot respectively in both cell lines and could be blocked by the anti-IL6 antibody Siltuximab. Three PCa cell lines, PC3, Du-145, and LNCaP-IL6+, with an autocrine IL6 loop displayed high expression of IRF9. A tissue microarray with 36 PCa tissues showed that IRF9 protein expression is moderately elevated in malignant areas and positively correlates with the tissue expression of IL6. Downregulation and overexpression of IRF9 provided evidence for an IFN-independent role of IRF9 in cellular proliferation of different PCa cell lines. Furthermore, expression of IRF9 was essential to mediate the antiproliferative effects of IFNα_2_. We concluded that IL6 is an inducer of IRF9 expression in PCa and a sensitizer for the antiproliferative effects of IFNα_2_.

## Introduction

According to the Global Cancer Statistics of 2011, prostate cancer (PCa) has the second highest incidence worldwide with 903 500 new cases in 2008 and 258 400 deaths (Jemal *et al*. 2011). Diagnosed in early stages, PCa is treated successfully either by surgery or by radiation therapy. In advanced stages of PCa, withdrawal of androgens is a standard therapy aimed at disease regression. However, in most cases, this therapy effect is transient, and the tumor relapses into a form of an aggressive castration-resistant PCa (CRPCa; Grossmann *et al*. 2001). Following the development of CRPCa, only palliative therapies are available including androgen receptor (AR) inhibition by non-steroidal anti-androgens, inhibition of intracrine androgen synthesis by abiraterone acetate, and inhibition of microtubule degradation by docetaxel. These therapies prolong survival only for about 2–3 months (Armstrong *et al*. 2010, Rane *et al*. 2012).

Androgen ablation induces T-cell infiltration of the prostate gland, thus leading to T-cell-mediated inflammation (Mercader *et al*. 2001). Inflammation processes are regulated by a network of cytokines that plays an important role in the interaction between cancer cells and the microenvironment. The pro-inflammatory cytokine interleukin 6 (IL6) has been identified as a regulatory factor for proliferation, apoptosis, angiogenesis, and differentiation (Akira *et al*. 1993). IL6 binds to the ligand-binding subunit gp80 of the IL6 receptor. The latter one interacts with the signal-transducing gp130 subunit, thus leading to the phosphorylation of Janus kinases. As a consequence, STAT3, phosphatidylinositol-3 kinase/Akt, and MAPK pathways are activated (Culig *et al*. 2005).

In several cancers, including PCa, IL6 is implicated in the development and progression of the disease (Trikha *et al*. 2003). High levels of IL6 protein correlate with advanced stages and poor prognosis (Siegall *et al*. 1990, Siegsmund *et al*. 1994, Adler *et al*. 1999, Drachenberg *et al*. 1999, Hobisch *et al*. 2000, Nakashima *et al*. 2000, Giri *et al*. 2001). *In vitro* studies demonstrated that IL6 treatment increases androgen receptor activity, thus leading to increased tumor cell proliferation or differentiation (Culig 2011). The anti-apoptotic protein MCL1 was shown to be positively regulated by IL6 and mediates the survival activity of IL6 (Cavarretta *et al*. 2007, 2008). IL6 increases resistance of PCa cells to the anti-androgen bicalutamide (Feng *et al*. 2009). Furthermore, data from a phase I study with the anti-IL6 chimeric MAB Siltuximab (CNTO328) demonstrated a downregulation of genes implicated in tumorigenesis (Karkera *et al*. 2011). Patients treated with Siltuximab revealed a decrease in phosphorylation of the IL6 downstream proteins STAT3 and p44/p42 MAPKs as well as enzymes responsible for intracellular androgen synthesis.

In this study, we aimed to identify novel genes implicated in IL6 signal transduction in PCa. For this purpose, we performed a gene expression microarray profiling upon treatment of two PCa cell lines, LNCaP and MDA PCa 2b, with 5 ng/ml IL6. We identified interferon (IFN) regulatory factor 9 (IRF9) as one of the commonly IL6-regulated genes in both cell lines. IRF9 has been demonstrated to mediate signaling of type I IFN (Darnell *et al*. 1994). We could show that IRF9 is regulated by IL6 and is highly expressed in cells with an autocrine IL6 loop. Furthermore, we showed that in our PCa cellular models, IRF9 exhibits a regulatory role in proliferation.

## Subjects and methods

### Cell lines and cell culture

The PCa cell lines MDA PCa 2b, LNCaP, PC3, and Du-145 were obtained from ATCC (Rockville, MD, USA). Cell line authenticity was confirmed by yearly short tandem repeat analysis. The LNCaP-derived cell line LNCaP-IL6+ was established by continuous treatment with 5 ng/ml IL6 (Biomedica, Vienna, Austria; Hobisch *et al*. 2001). LNCaP, PC3, and Du-145 cells were cultured in RPMI-1640 medium containing 10% FCS and 20 mmol/l glutaMax (both from PAA Laboratories, Pasching, Austria). The F-12 Ham Kaighn's modification medium for the MDA PCa 2b cell line was supplemented with 20% FCS, 25 ng/ml cholera toxin (Sigma–Aldrich), 10 ng/ml mouse epidermal growth factor (GroPep, Adelaide, BC, Australia), 0.005 mM phosphoethanolamine (Sigma–Aldrich), 100 pg/ml hydrocortisone (Sigma–Aldrich), 0.125 mg/ml selenious acid (Sigma–Aldrich), 0.095 mg/ml human transferrin (Sigma–Aldrich), and 0.105 mg/ml human insulin (Sigma–Aldrich) as recommended by ATCC.

MDA PCa 2b and LNCaP cells were seeded onto poly-d-lysine hydrobromide (30 mg/ml; Sigma–Aldrich) pre-coated plates. LNCaP cells were seeded at a density of 10^4^ cells/well and LNCaP-IL6+ and PC3 cells were seeded at a density of 2×10^3^ cells/well onto 96-well plates. MDA PCa 2b and LNCaP cells were seeded at a density of 5×10^5^ cells and LNCaP-IL6+, PC3, and Du-145 cells at a density of 3.5×10^5^ cells/well onto six-well plates. The cells were starved with medium containing 3% dextran charcoal-stripped FCS (CS FCS, Thermo Fisher Scientific, Inc., Waltham, MA, USA) and 20 mmol/l glutaMax for 24 h. All experiments were performed in a medium supplemented with 3% CS FCS and 20 mmol/l glutaMax. Treatment with 5 ng/ml IL6, 50 μg/ml Siltuximab, or 1000 U/ml IFNα_2_ was performed 24 h after changing the medium.

### [^3^H]thymidine incorporation assay

[^3^H]thymidine incorporation assays were performed in 96-well plates as described earlier (Hoefer *et al*. 2012). Each experiment was seeded in quintuplicates. For downregulation experiments, the cells were treated with 50 nmol/l siRNA against IRF9 or control siRNA for 72 h. For overexpression experiments, transfection with 0.1 μg/ml per well pcDNA3-HA-IRF9 or pcDNA3 was performed for 72 h.

### Western blotting and IL6 measurements

Western blot experiments were performed in six-well plates. For downregulation experiments, the cells were transfected with 50 nmol/l siRNA against IRF9 or control siRNA for 48 h. For overexpression experiments, transfection with 1 μg/ml per well pcDNA3-HA-IRF9 or pcDNA3 was performed for 48 h. Western blot was performed as described earlier (Cavarretta *et al*. 2007). The following antibodies were used: anti-IRF9 (1:1000, Sigma–Aldrich), anti-glyceraldehyde-3-phosphate dehydrogenase (1:50 000, Chemicon International, Inc., Billerica, MA, USA), and anti-lamin A (1:1000, Abcam, Cambridge, UK).

IL6 levels were determined in the supernatants obtained after 24-h culture in serum-free medium of PC3 and LNCaP-IL6+ cells using an immunoassay by the Central Institute for Medical and Chemical Laboratory Diagnostics, Innsbruck Medical University.

### Affymetrix gene expression microarray

LNCaP and MDA PCa 2b cells were seeded in six wells each. The cells were starved for 24 h and were treated with 5 ng/ml IL6 for 18 h. Both cell lines were seeded in triplicates and two biological replicates were performed, resulting in 12 samples per cell line. Cells were lysed using RLT buffer (Qiagen) and homogenized using the QIAshredder spin column (Qiagen). RNA was isolated using the miRNeasy 96 kit by following the manufacturer's instructions (Qiagen). During RNA isolation, an on-plate DNase digestion was performed. The RNA concentration of the samples was determined on a Nanodrop-8000 u.v.–vis Spectrophotometer (Thermo Scientific, Waltman, MA, USA). RNA integrity was determined with the RNA 6000 Nano Kit on an Agilent 2100 Bioanalyzer (Agilent Technologies, Inc., Santa Clara, CA, USA).

Biotin-labeled, amplified RNA (aRNA) was synthesized from 200 ng total RNA using the 3′IVT Express Kit (Affymetrix, Santa Clara, CA, USA). The aRNA was purified using Agencourt RNAClean XP beads (Beckman Coulter, Inc., Indianapolis, IN, USA) on the BioMek Fx Workstation (Beckman Coulter, Inc.). Biotin-labeled aRNA was fragmented using the 3′IVT Express Kit (Affymetrix). The fragmented biotin-labeled aRNA (4.5 μg) was hybridized on a HT Human Genome U133 96-Array plate (Affymetrix). The 96-array plate was washed, stained, and scanned with the GeneTitan Instrument (Affymetrix).

The microarray data were normalized with RMA (Irizarry *et al*. 2003) and summarized with the hthgu133pluspmhsentrezgcdf_15.0.0 CDF (Dai *et al*. 2005). Genes that show little expression variation across the samples are filtered based on I/NI-calls (Talloen *et al*. 2007), leaving 5934 of the 18 837. Linear models for microarray data analysis (Limma, Seattle, WA, USA) were used to identify genes that are changed upon treatment (Smyth *et al*. 2003).

### Tissue microarray and immunohistochemistry

For the construction of a tissue microarray (TMA) for evaluation of IRF9 and IL6 expression, formalin-fixed, paraffin-embedded tissue samples displaying high amounts of tissue-infiltrating lymphocytes were selected. The clinical demographics of this cohort were as follows: 36 PCa tissues; Gleason score (GSC)<7: *n*=7, GSC=7: *n*=23, GSC>7: *n*=6; patients' age, mean±s.d., 62±6 years; patients' serum prostate-specific antigen level, 5±3.1 ng/ml; prostate volume, 48±16.4 g. For each case, three cancer tissue cores and three benign cores were punched. The TMA was assembled using a manual tissue arrayer (Beecher Instruments, Sun Prairie, WI, USA). For control of histological diagnosis, cytokeratin 5/6 and α-methylacyl-CoA racemase IHC double stainings were performed on a Discovery-XT staining device (Ventana, Tucson, AZ, USA). The use of archived samples deriving from radical prostatectomy was approved by the Ethics Committee of Innsbruck Medical University. Staining and evaluation of the samples were performed as described earlier (Karkera *et al*. 2011). The following antibodies were used: anti-IRF9 (1:50, Sigma–Aldrich) and anti-IL6 (ab6672, 1:100, Abcam).

### Transfections

For siRNA transfections, 50 nmol/l ON-TARGETplus SMARTpool against IRF9 or against IL6 (both Dharmacon, Thermo Scientific, Vienna, Austria) were used. As a control, the ON-TARGETplus Non-targeting Control Pool was used (Dharmacon, Thermo Scientific). Transfections of siRNA were performed with Lipofectamine 2000 (Invitrogen) according to the manufacturer's protocol. For overexpression experiments, the plasmid pcDNA3-HA-IRF9 (Addgene plasmid 11614 from Steven Johnson Lab) and the empty vector pcDNA3 (Invitrogen) were used. Depending on the well size, the cells were transfected with 1 μg/ml plasmid in six-well plates and 0.1 μg/ml in 96-well plates using X-tremeGENE HP DNA transfection reagent (Roche) for 48 or 72 h following the manufacturer's instructions.

### Nuclear/cytoplasmatic fractionation

Nuclear/cytoplasmatic fractionation was performed with the NE-PER nuclear and cytoplasmatic extraction kit (Pierce, Vienna, Austria) by following the manufacturer's instructions.

### RNA isolation and quantitative real-time PCR

Quantitative real-time PCR (QRT-PCR) experiments were performed in six-well plates. Cells were harvested after treatment with 5 ng/ml IL6 for 18 h. RNA was isolated using the RNeasy mini kit by following the manufacturer's instructions (Qiagen). cDNA synthesis was performed using iScript select cDNA synthesis kit (Bio-Rad). QRT-PCR was performed using the ABI Prism 7500 Fast RT-PCR System and TaqMan gene expression assays for IRF9 (both Applied Biosystems). HPRT1 was used as a control. ABI Sequence Detection Software was used for determination of *C*_t_ values. 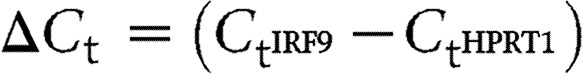 values were calculated and gene regulation expressed as 
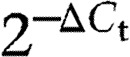
.

### Statistical analysis

All experiments have been performed in at least three biological replicates. Pearson correlations were calculated using IBM SPSS Statistics (IBM). Student's *t*-test was used to assess significant differences between the control and the indicated treatment group. Significances are encoded as follows: *P≤0.05; **P≤0.01; ***P≤0.001.

## Results

### IL6 regulates IRF9 expression in LNCaP and MDA PCa 2b cells

**Figure 1:**
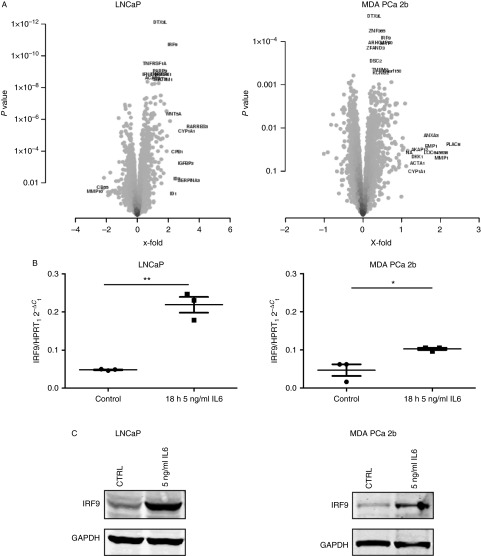
Identification of IRF9 as an IL6-regulated gene in LNCaP and MDA PCa 2b cells. (A) LNCaP and MDA PCa 2b cells were treated for 18 h with 5 ng/ml IL6 and profiled on Affymetrix microarrays. These volcano plots show the results of a test for differential expression between IL6-treated and untreated cells, with the significance (*P* value) in the *Y*-axis and the log fold change in the *X*-axis. IRF9 is among the top genes with the smallest *P* values regulated by IL6 in both cell lines. (B) To validate results from the microarray experiment, LNCaP and MDA PCa 2b cells were treated with 5 ng/ml IL6 for 18 h and QRT-PCR was performed. Values indicated are mean±s.e.m., *n*=3. (C) Protein regulation of IRF9 through IL6 treatment (5 ng/ml for 48 h) was controlled by western blotting. A representative western blot of three independent experiments is shown. *P<0.05; **P<0.01.

To identify novel genes regulated by IL6 in PCa cell lines, we cultured LNCaP and MDA PCa 2b cells for 18 h in the presence or absence of 5 ng/ml IL6 and performed gene expression profiling on Affymetrix microarrays. The data have been submitted to GEO database (accession number GSE47973). We used a two-group limma comparison in both cell lines to identify genes that are regulated by IL6 treatment. We detected substantial gene expression changes induced in the LNCaP cell line, where 672 genes are significant at adjusted *P* value ≤0.05. No genes met this criterion for MDA PCa 2b cell line; however, 931 genes were found to be significantly differentially expressed at *P* value ≤0.05. The volcano plot in Fig. 1A shows the fold changes and *P* values of all genes. The genes with the most significant *P* values and/or the largest fold changes are depicted with their names. The top genes regulated by IL6 according to the *P* values are listed in Table 1. For further investigation, we selected IRF9 as the gene regulated by IL6 in both LNCaP and MDA PCa 2b. To confirm the IL6 regulation of IRF9 in LNCaP and MDA PCa 2b cells, we performed QRT-PCR analysis. As shown in Fig. 1B, IRF9 was found to be significantly increased in IL6-treated LNCaP and MDA PCa 2b cells. Additionally, we performed western blot analysis to confirm that IL6 also increases the protein levels of IRF9. When exposed to IL6 for 48 h, both cell lines showed an increase in IRF9 protein expression (Fig. 1C). Altogether, we concluded that under our experimental conditions, IL6 upregulates IRF9 in LNCaP and MDA PCa 2b cells at the mRNA and protein levels.

### IRF9 is highly expressed in IL6-producing PCa cell lines

**Figure 2:**
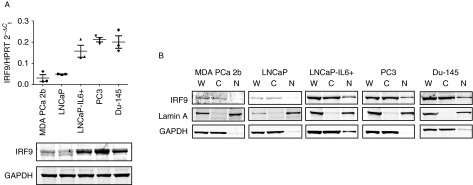
Expression and localization of IRF9 in prostate cancer (PCa) cell lines. (A) mRNA levels were measured by QRT-PCR and normalized to *HRPT1* mRNA in different PCa cell lines and were plotted as mean±s.e.m. of three independent experiments. IRF9 expression was assessed by western blotting. One representative of three independent experiments is shown. (B) Localization of IRF9 in PCa cell lines was determined by western blot after nuclear/cytoplasmatic fractionation. One representative western blot of three independent experiments is shown. W, whole cell lysate; C, cytoplasm; N, nuclei.

The cell lines LNCaP-IL6+, PC3, and Du-145 express high levels of IL6 mRNA (Supplementary Figure S1, see section on supplementary data given at the end of this article) and protein expression (Okamoto *et al*. 1997), while LNCaP and MDA PCa 2b cells have low levels of *IL6* mRNA expression (Supplementary Figure S1) and no detectable IL6 secretion (data not shown). To address the question whether IRF9 expression is elevated in the IL6-producing cell lines, QRT-PCR and western blot were performed (Fig. 2A). Indeed, high IRF9 mRNA and protein expression levels could be observed in LNCaP-IL6+, PC3, and Du-145 cell lines, leading to the conclusion that the autocrine production of IL6 is sufficient to upregulate IRF9 expression. A nuclear localization sequence has been detected in IRF9 (Reich & Liu 2006), enabling its shuttling to the nucleus in the complex with STAT factors. To address the question whether IRF9 is also present in the nucleus of PCa cell lines, nuclear/cytoplasmatic fractionation assays were performed. We observed a predominantly cytoplasmatic localization in the tested cell lines (Fig. 2B). However, it could not be excluded that a small proportion of IRF9 is present in the nuclei, especially in the IL6-producing cell lines LNCaP-IL6+, PC3, and Du-145.

### IRF9 expression in human prostate tissue

**Figure 3:**
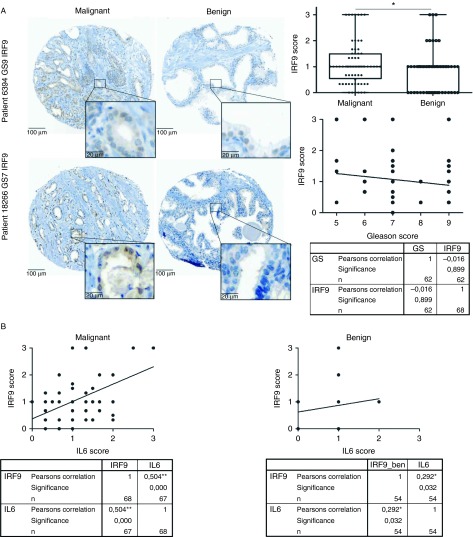
IRF9 expression in human prostate tissue. (A) Immunohistochemical staining of one representative benign and one malignant core from two prostate cancer (PCa) patients for IRF9 and evaluation of staining intensity of the PCa TMA. (B) Immunohistochemical staining of IL6 was performed on a consecutive slice of the TMA. Expression of IL6 was correlated with that of IRF9 in benign and malignant cores of PCa patients. *P<0.05; **P<0.01.

To investigate IRF9 expression in prostatic cancerous tissue, we performed a TMA with tissues from 36 PCa patients. This included specimens with a high infiltration of macrophages in cancerous lesions as described in the Subjects and methods section. Specificity of the IRF9 antibody was controlled using PC3 cells transduced with pcDNA3-HA-IRF9 or siIRF9 (Supplementary Figure S2A, see section on supplementary data given at the end of this article). The IRF9 expression patterns showed a very heterogeneous distribution between the different samples ranging from negative to very high expression in both benign and malignant areas (Fig. 3A). Statistical analysis of the TMA demonstrated that the average staining intensity of IRF9 in tumors compared with benign tissue marginally but statistically significantly increased. However, no significant correlation of IRF9 staining with Gleason score was found (Fig. 3A). Furthermore, staining for IL6 was performed on a consecutive slice of the TMA. Again, specificity of the IL6 antibody was controlled using IL6-negative LNCaP and IL6-positive PC3 cells (Supplementary Figure S2B). Staining patterns for IL6 were consistent with those previously observed (Hobisch *et al*. 2000; Supplementary Figure S3A). Staining intensity for both IRF9 and IL6 increased in benign and, marginally, in malignant cores with a high inflammatory index (Supplementary Figure S3B and C). Interestingly, the staining scores of IRF9 showed a moderate correlation (Pearson correlation coefficient 0.504; *P*<0.01) with the staining pattern of IL6 in malignant areas (Fig. 3B). On the other hand, no such correlation could be found in benign areas (Pearson correlation coefficient 0.292; *P*<0.05). We concluded that IL6 expressed in malignant areas of the prostate could be an inducer of IRF9 expression.

### Siltuximab counteracts IRF9 regulation through IL6 in LNCaP and MDA PCa 2b

**Figure 4:**
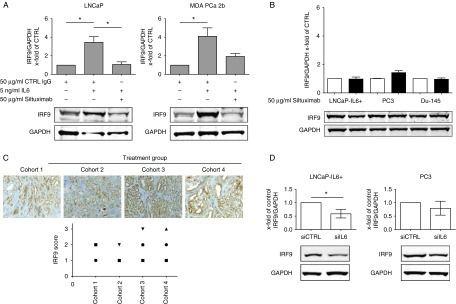
Siltuximab counteracts the IL6-induced expression of IRF9 in LNCaP and MDA PCa 2b, but not in IL6-producing prostate cancer (PCa) cell lines or PCa tissue. (A) LNCaP and MDA PCa 2b cells were treated with 50 μg/ml control IgG, 5 ng/ml IL6, or 50 μg/ml Siltuximab for 48 h, as indicated. (B) IL6-producing LNCaP-IL6+, Du-145, and PC3 cells were treated with 50 μg/ml Siltuximab or control IgG for 48 h. (A and B) IRF9 expression was assessed by western blot (one representative blot is shown). Band intensity was quantified and mean±s.d., *n*=3 is shown. (C) Immunohistochemical staining for IRF9 of representative cores from PCa patients from a clinical phase 1 study with Siltuximab and evaluation of the staining intensity. Cohort 1: placebo group. Cohorts 2, 3, and 4: one, two, or three injections of Siltuximab (6 mg/kg, 29, 15, and 1 day prior to radical prostatectomy), respectively. (D) Representative western blot of IRF9 expression after 72-h downregulation of IL6. Band intensity was quantified and mean±s.d., *n*=3 is shown. *P<0.05.

It was previously reported that exogenous IL6 activity can be neutralized by the chimeric anti-IL6 antibody Siltuximab (Li *et al*. 2005). Therefore, we questioned whether the IL6 regulation of IRF9 can be inhibited by cotreatment of IL6 and Siltuximab. We found that in LNCaP and MDA PCa 2b cells cotreated with IL6 and Siltuximab, IRF9 expression returned almost to basal levels when compared with untreated cells (Fig. 4A). Addition of IL6 did not have any additional effect on IRF9 expression in the IL6-secreting LNCaP-IL6+, PC3, and Du-145 cell lines (Supplementary Figure S4A, see section on supplementary data given at the end of this article). Interestingly, treatment of those cells with Siltuximab did not reduce IRF9 protein expression, leading to the conclusion that extracellular inhibition of IL6 signaling is insufficient to reduce IRF9 expression (Fig. 4B).

To further corroborate those findings, we analyzed *in situ* IRF9 expression in malignant cores of 19 PCa patients from a phase I study that was previously performed (Karkera *et al*. 2011; Fig. 4C). In this study, cohort 1 served as a control group as those patients did not receive any treatment. Patients in cohorts 2, 3, and 4 were treated with one, two, or three injections of Siltuximab (6 mg/kg, 29, 15, and 1 day prior to surgery) respectively. Evaluation of stainings revealed no significant difference in IRF9 expression in treated (cohorts 2–4) patients compared with the untreated group (cohort 1). Next, we analyzed whether inhibition of IL6 signaling by downregulation of IL6 expression could decrease IRF9. Indeed, we could find a decreased IRF9 expression in PC3 and LNCaP-IL6+ cells treated with siRNA against IL6 (Fig. 4D and Supplementary Figure S4B).

### Differential effects of IRF9 on cellular proliferation

**Figure 5:**
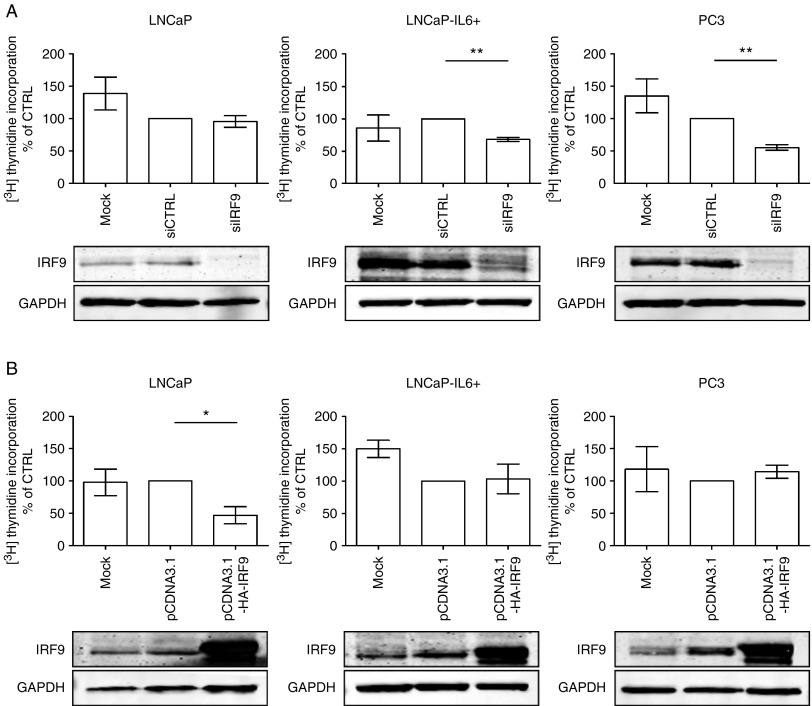
Modulation of IRF9 expression influences proliferation of prostate cancer cell lines. (A and B) Proliferation was measured by [^3^H]thymidine incorporation assay after downregulation and overexpression of IRF9 after 72 h in LNCaP, LNCaP-IL6+, and PC3 cells, as indicated. Data represent mean±s.e.m. from five independent experiments. Downregulation (A) and overexpression (B) of IRF9 was controlled by western blot. *P<0.05; **P<0.01.

IRF9 is implicated in modulation of STAT1 signaling, which is known to play a role in proliferation and apoptosis in PCa (Frank 1999, 2003). To address the question whether IRF9 is involved in PCa cell proliferation, we performed [^3^H]thymidine incorporation assays after downregulation of IRF9 by a siRNA approach. Efficient downregulation of IRF9 in LNCaP, LNCaP-IL6+, and PC3 cells was controlled by western blotting (Fig. 5A). In LNCaP cells, which express very low levels of IRF9 and are inhibited by IL6 (Hobisch *et al*. 2001), depletion of IRF9 by siRNA did not have any effect on proliferation when compared with the siRNA control. In contrast, downregulation of IRF9 significantly reduced cell proliferation by ∼30–50% in LNCaP-IL6+ and PC3 cells. We concluded that IRF9 has a pro-proliferative role in cells that have high IRF9 expression.

To further corroborate a possible pro-proliferative role of IRF9, we transfected LNCaP, LNCaP-IL6+, and PC3 cells with the pcDNA3-HA-IRF9 expression vector or the pcDNA3 control vector for 72 h and determined proliferation by [^3^H]thymidine incorporation. Surprisingly, overexpression of IRF9 significantly decreased proliferation to almost 50% in LNCaP cells but did not affect proliferation of LNCaP-IL6+ and PC3 cells (Fig. 5B). Thus, we concluded that IRF9 has differential effects on proliferation of PCa cell lines depending on the physiological state of the cells.

### IRF9 downregulation antagonizes the antiproliferative effect of IFNα_2_

**Figure 6:**
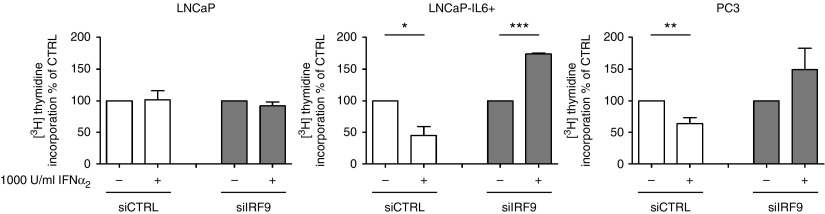
IRF9 mediates the antiproliferative effects of IFNα_2_. Proliferation was measured by [^3^H]thymidine incorporation assay in LNCaP, LNCaP-IL6+, and PC3 cells after downregulation of IRF9 and additional treatment with 1000 U/ml IFNα_2_ after 72 h. Data represent mean±s.e.m. from three independent experiments. *P<0.05; **P<0.01; ***P<0.001.

Previous studies have shown that IRF9 plays an important role in IFNα_2_ signaling and mediates the antiproliferative effect of IFNα_2_ (Caraglia *et al*. 2005, Tsuno *et al*. 2009). Furthermore, it has been reported that IFNα_2_ decreases proliferation of PCa cells and several other tumors *in vitro* and *in vivo* and IFNα_2_ treatment is one of the few FDA-approved cancer immunotherapeutics (Hobeika *et al*. 1999, Kirkwood 2002, Bracarda *et al*. 2010). For this reason, we tested whether IRF9 plays a role in the antiproliferative effects of IFNα_2_. We transfected LNCaP, LNCaP-IL6+, and PC3 cells with IRF9 or control siRNA, treated the cells with 1000 U/ml IFNα_2_, and measured proliferation after 48 h by [^3^H]thymidine incorporation. LNCaP transfected with IRF9 or control siRNA showed no change in proliferation after IFNα_2_ treatment, whereas LNCaP-IL6+ and PC3 cells transfected with control siRNA showed a significant decrease in proliferation after IFNα_2_ treatment (Fig. 6). However, proliferation of *IRF9* siRNA-transfected LNCaP-IL6+ and PC3 cells increased after IFNα_2_ treatment compared with the untreated *IRF9* siRNA-transfected cells. This shows that high expression of IRF9 through IL6 signaling is needed to transmit the antiproliferative effects of IFNα_2_.

## Discussion

In this work, we were able to demonstrate that IRF9 mRNA and protein expression is regulated by IL6 in LNCaP and MDA PCa 2b cells. Weihua *et al*. (2000) first reported on IL6-induced IRF9 in HeLa cells. It was found that IL6 induces transcription of IRF9 by C/EBP-β binding to the γ-IFN-activated transcriptional element. Thus, IL6 is able to prime cells for the response to IFNα_2_. However, the pathophysiological role of the IRF9 protein in human cancer has not been completely clarified yet. Stimulation with type I IFN leads to the formation of the transcriptionally active complex IFN-stimulated gene factor 3 (ISGF3) consisting of IRF9, STAT1, and STAT2 (Darnell *et al*. 1994). The ISGF3 complex shuttles to the nucleus where it acts as a transcription factor to mediate the expression of IFN-stimulated genes. Tsuno *et al*. (2009) showed that IRF9 is responsible for the antiproliferative effects of IFNα_2_ in human ovarian adenocarcinoma. Previously, it was demonstrated that IFNα_2_ may decrease proliferation by increasing the p21 cell cycle inhibitor, thus leading to a cell cycle arrest (Sica *et al*. 1989, Hobeika *et al*. 1997). Here, we could show that IRF9 is needed to transmit the antiproliferative effects of IFNα_2_ in cells with high IL6 and IRF9 expression (Fig. 6). By contrast, in LNCaP cells that have absent/low expression of IL6 and IRF9, IFNα_2_ treatment lacks antiproliferative effects. Thus, we conclude that the antiproliferative effect of IFNα_2_ signaling is dependent on IL6-induced IRF9 expression in PCa cell lines. We have also addressed the question whether IRF9 has an effect on proliferation without IFNα_2_ treatment. Opposite effects were found after modulating IRF9 expression in LNCaP vs LNCaP-IL6+ and PC3 cells (Fig. 5A and B). It is therefore hypothesized that IRF9 has additional cellular functions besides its role in mediating IFNα_2_ signaling. Indeed, it was previously demonstrated that upregulated IRF9 confers resistance to the antimicrotubule agent paclitaxel in drug-resistant breast cancer cells (Luker *et al*. 2001). Importantly, paclitaxel resistance could not be induced by IFNα_2_ treatment. On the other hand, IL6 is a known inducer of paclitaxel resistance in breast cancer (Conze *et al*. 2001). Thus, IL6-dependent upregulation of IRF9 could be an important mechanism for paclitaxel resistance in breast cancer or for docetaxel resistance in PCa. Future clinical trials may consider the fact that IL6 is an important regulator of IRF9 expression. Interpretation of a clinical study may depend on the stage of the disease when the treatment with an anti-IL6 antibody is initiated. Indeed, also STAT1, a member of the ISGF3 complex, was found to be increasingly expressed in docetaxel-resistant PCa cell lines (Patterson *et al*. 2006). This shows that IRF9 has IFNα_2_-independent functions, which are worth to be investigated in more detail.

IFNα_2_ is used to treat patients with several malignant diseases such as hairy cell leukemia, lymphoma, renal cell carcinoma, bladder cancer, breast cancer, and melanoma (Borden 1984, Kirkwood 2002). In clinical studies with IFNα_2_ in PCa, only four of ten patients responded to the treatment and one patient showed a reduction in bone pain (Gutterman & Quesada 1981, Chang *et al*. 1986). However, it was demonstrated that the concentrations of recombinant IFNα_2_ used (5–10×10^6^ U/m^2^, three times a week) lead to an intolerable toxicity in patients with advanced prostate carcinoma (Chang *et al*. 1986). Recently, pegylated IFNα_2_ (IFN molecules with a covalently attached polyethylene glycol moiety), a more stable form of IFNα_2_, has been developed and tested in clinical studies. However, toxicity of pegylated IFNα_2_ is still an issue (Daud *et al*. 2012). We have demonstrated that the expression of IRF9 is a requisite for transmitting the antiproliferative effects of IFNα_2_ in IL6 producing PCa cells (Fig. 6). On the other hand, the staining patterns of IRF9 in PCa specimen were heterogeneous but correlated with IL6 expression in the tissue (Fig. 3). Thus, our data suggest that clinical studies with administration of IFNα_2_ should consider the expression of IRF9 in the cancerous tissue in order to select patients that could benefit from such a therapy known to have severe toxic side effects. In general, high expression of IL6 in tissue is associated with bad prognosis of PCa patients (Ishiguro *et al*. 2011). However, as IL6 is a potent inducer of IRF9, high IL6 expression could possibly serve in this case as an indicator for a favorable response to IFNα_2_ treatment. In clinics, IFN treatment is often given as adjuvant therapy. Interestingly, experiments with pegylated IFNα_2_ combined with docetaxel in mice bearing orthotopic implantations of PC3-MM2 PCa cells resulted in a reduction of tumor weight and increased apoptosis of tumor-associated endothelial cells (Huang *et al*. 2002). Another study showed that low concentrations of IFNα_2_ increased the cytotoxic effects of the histone deacetylase-inhibitor valproic acid on PCa cells (Hudak *et al*. 2012). However, expression of IRF9 was not addressed in these studies.

In summary, we show that IRF9 protein expression is increased in human PCa and correlates with IL6 expression in cancer areas. The antiproliferative effect of IFNα_2_ is dependent on the expression of IRF9 and IFNα_2_-independent functions of IRF9 have been observed. On the basis of these results, we suggest that IRF9 may be considered as a marker for IFNα_2_ therapies in PCa.

## Supplementary data

This is linked to the online version of the paper at http://dx.doi.org/10.1530/ERC-13-0222.

## Author contribution statement

H H H Erb performed all *in vitro* experiments in cell lines, analyzed all data, planned immunohistochemical experiments with patients' specimen, and wrote the first draft of the paper. R V Langlechner investigated the expression of IRF9 in prostate cancer cell lines and analyzed the data. P L Moser planned and supervised immunohistochemical data and analyzed the expression of IRF9 in patients' material. F Handle investigated the functional relevance of IRF9 in cell lines and analyzed the data. T Casneuf performed microarrays and analyzed the data. K Verstraeten performed the Affymetrix gene expression microarray analysis. B Schlick was involved in selection of patients for tissue microarray. G Schäfer was involved in selection of patients for tissue microarray and analyzed inflammation status for tissue microarray. B Hall participated in the design of the experiments, supervised microarrays, and analyzed the data. K Sasser coordinated research at Janssen R&D and analyzed the data. Z Culig designed the study, supervised and coordinated research, analyzed data, and corrected the first draft of the manuscript. F R Santer is responsible for day-to-day supervision, coordinated research, analyzed data, and corrected the first draft of the manuscript. Z Culig and F R Santer are joint senior authors.

## Supplementary Material

Supplementary data

## Figures and Tables

**Table 1:** IL6-regulated genes in prostate cancer cell lines. List of top ten genes regulated in LNCaP and MDA PCa 2b cell lines after treatment for 18 h with 5 ng/ml IL6. Regulation (LogFC) and significance (adjusted *P* values (Adj.*P*.val)) are indicated.

**Symbol**	**Gene name**	**LogFC**	**Adj.*P*.val**
LNCaP			
*DTX3L*	Deltex 3-like (*Drosophila*)	1.450404539	4.48×10^−9^
*IRF9*	Interferon regulatory factor 9	2.246350245	5.69×10^−8^
*TNFRSF1A*	Tumor necrosis factor receptor superfamily, member 1A	1.107492748	5.90×10^−7^
*PARP9*	Poly (ADP-ribose) polymerase family, member 9	1.436627837	1.26×10^−6^
*HMOX1*	Heme oxygenase (decycling) 1	1.702377153	1.26×10^−6^
*TNFAIP8*	Tumor necrosis factor, alpha-induced protein 8	1.410625072	1.26×10^−6^
*IFNAR1*	Interferon (alpha, beta, and omega) receptor 1	0.848829894	1.26×10^−6^
*ACAD8*	Acyl-CoA dehydrogenase family, member 8	0.97607392	1.79×10^−6^
*STAT3*	Signal transducer and activator of transcription 3 (acute-phase response factor)	1.443006989	1.79×10^−6^
*SECTM1*	Secreted and transmembrane 1	1.692340363	1.82×10^−6^
MDA PCa 2b			
*DTX3L*	Deltex 3-like (*Drosophila*)	0.2835761	0.129023885
*ZNF365*	Zinc finger protein 365	0.361045312	0.129023885
*IRF9*	Interferon regulatory factor 9	0.588743511	0.129023885
*ARHGAP10*	Rho GTPase activating protein 10	0.443541337	0.129023885
*MVP*	Major vault protein	0.583323921	0.129023885
*ZFAND3*	Zinc finger, AN1-type domain 3	0.322167608	0.138694101
*DSC2*	Desmocollin 2	0.327017732	0.225034496
*TMEM2*	Transmembrane protein 2	0.443119481	0.225034496
*C9orf150* (*LURAP1L*)	Chromosome 9 open reading frame 150	0.758516083	0.225034496
*KCND2*	Potassium voltage-gated channel, Shal-related subfamily, member 2	0.459400324	0.225034496
