# Supplementary material for: IL6 sensitizes prostate cancer to the antiproliferative effect of IFNα2 through IRF9
Source: Endocr Relat Cancer. 2013 Aug 2;20(5):677–89. doi: 10.1530/ERC-13-0222 (PMC3753051; doi:10.1530/ERC-13-0222)
Supplement: Supplementary data [file supp_20_5_677__index.html]

Supplementary data 

# IL6 sensitizes prostate cancer to the antiproliferative effect of IFNα2 through IRF9

## Supplementary data

**Files in this Data Supplement:**

- Supplementary figure 1 - IL-6 mRNA expression of LNCaP, MDA PCa 2b, LNCaP IL-6+, PC3, and Du-145 cells was assessed by QRT-PCR. IL-6 mRNA expression was normalized to HPRT1 and is shown as mean ± SEM from three independent experiments. (PDF 20 KB)
- Supplementary figure 2 - (A) IRF9 antibody specificity for immunohistochemical experiments was controlled using formalin-fixed paraffin-embedded untransfected PC3 or PC3 transfected with pcDNA-HA-IRF9 or siIRF9, as indicated. (B) IL-6 antibody specificity for immunohistochemical experiments was controlled using formalin-fixed paraffin-embedded LNCaP (IL-6 negative) or PC3 (IL-6 positive). (PDF 997 KB)
- Supplementary figure 3 - (A) Immunohistochemical staining of two representative benign and malignant cores of a prostate cancer patient for IL-6 and evaluation of staining intensity of the prostate cancer TMA. (B, C) Patient cores were classified in low and high inflammation groups based on low and high infiltration of macrophages. IRF9 (B) and IL-6 (C) stainings were correlated with the two groups. (PDF 1,208 KB)
- Supplementary figure 4 - (A) IRF9 mRNA expression of LNCaP, MDA PCa 2b, LNCaP-IL-6+, PC3, and Du-145 cells was assessed after treatment with 5 ng/ml IL-6 for 18 h by QRT-PCR. IRF9 mRNA expression was normalized to HPRT1 and is shown as mean ± SEM from three independent experiments. (B) Secreted IL-6 was measured after 24h in serum-free supernatants of PC3 and LNCaP-IL-6+ cells 72 h post-transfection with control or IL-6 siRNA, as indicated. (PDF 940 KB)
