# Supplementary figures and images for: IL6 sensitizes prostate cancer to the antiproliferative effect of IFNα2 through IRF9
Source: Endocr Relat Cancer. 2013 Aug 2;20(5):677–89. doi: 10.1530/ERC-13-0222 (PMC3753051; doi:10.1530/ERC-13-0222)

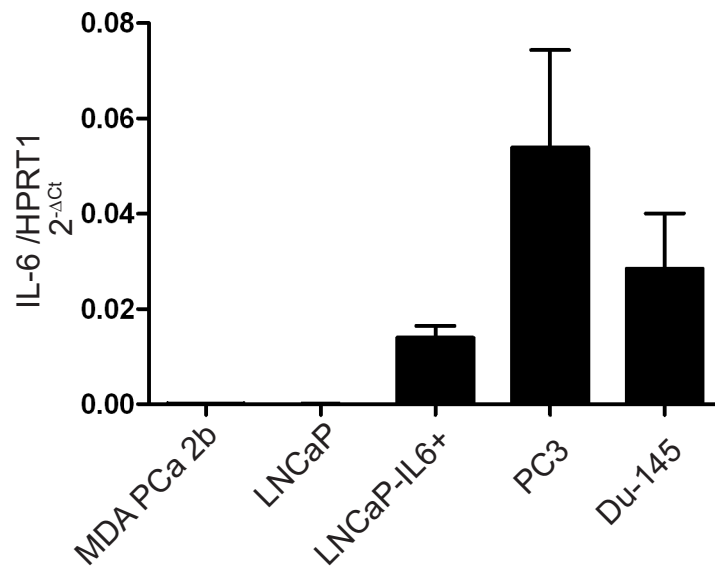

Fig.S1 Erb et al., 2013

Supplement: Supplementary data [file supp_ERC-13-0222_Supplementary_figure_1.pdf]

**A**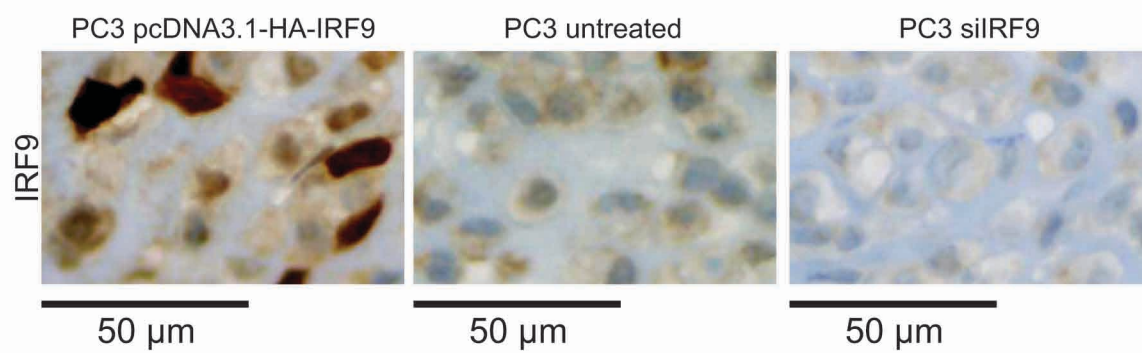**B**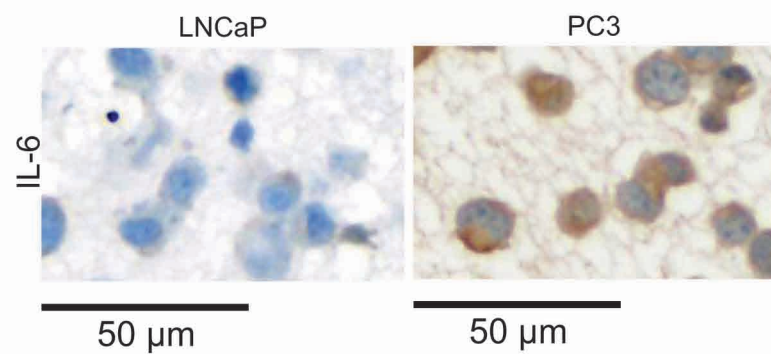

Supplement: Supplementary data [file supp_ERC-13-0222_Supplementary_figure_2.pdf]

**A**

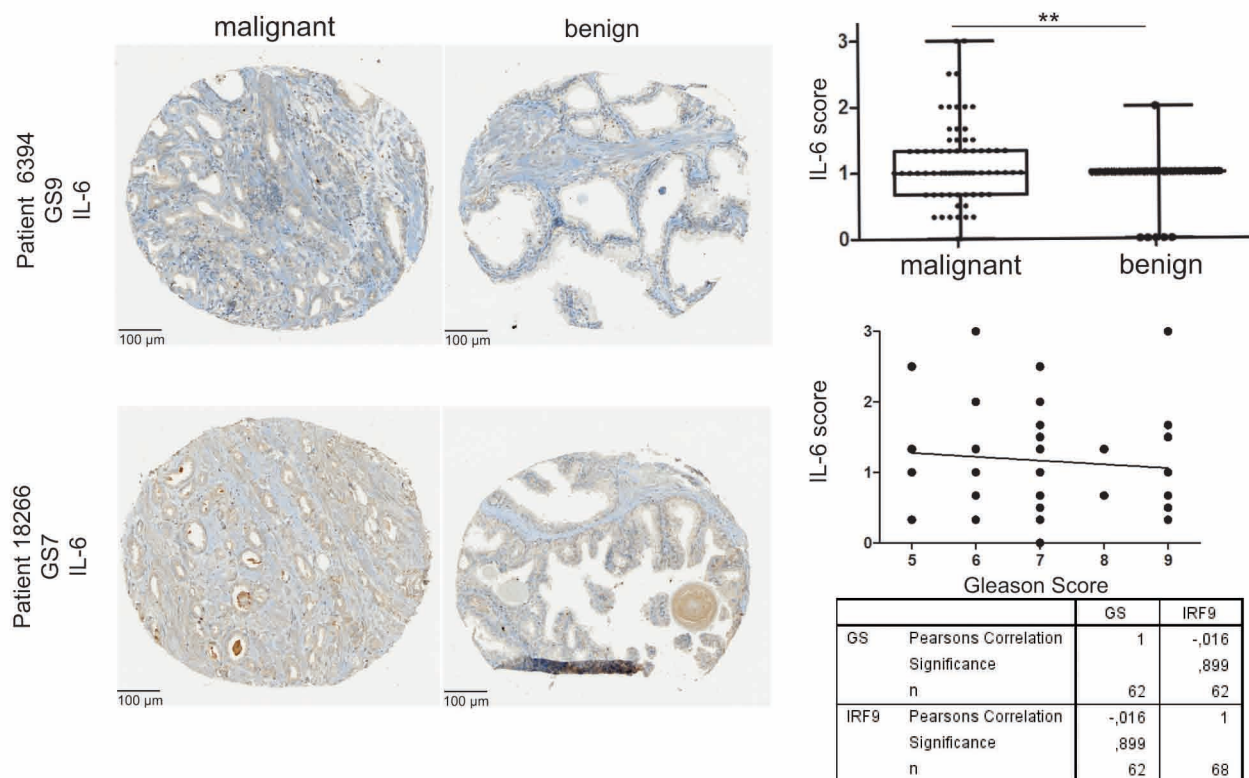

**B**

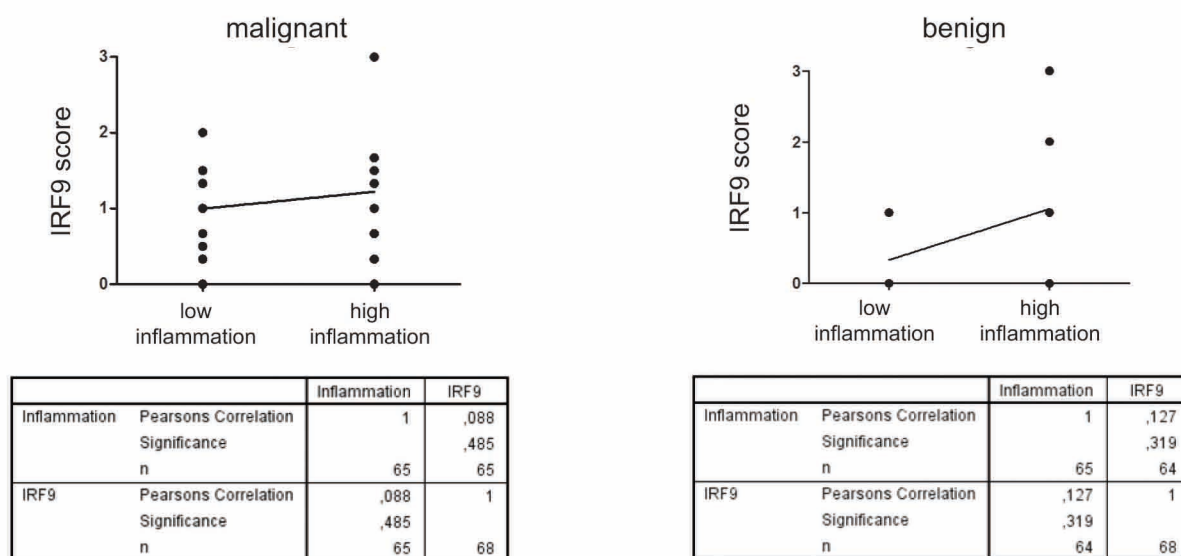

**C**

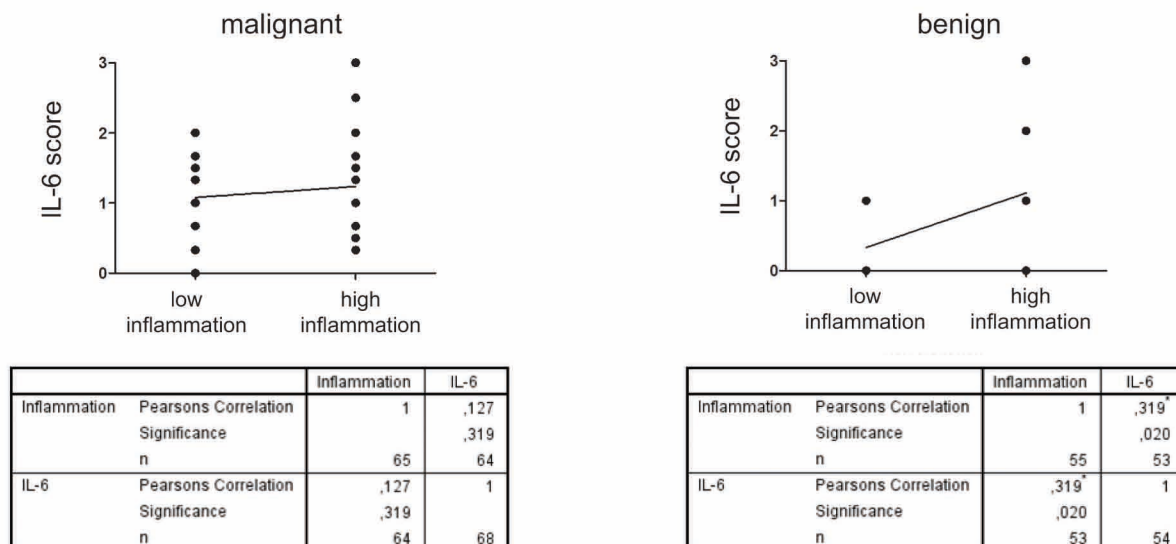

Fig.S3 Erb et al., 2013

Supplement: Supplementary data [file supp_ERC-13-0222_Supplementary_figure_3.pdf]

**A**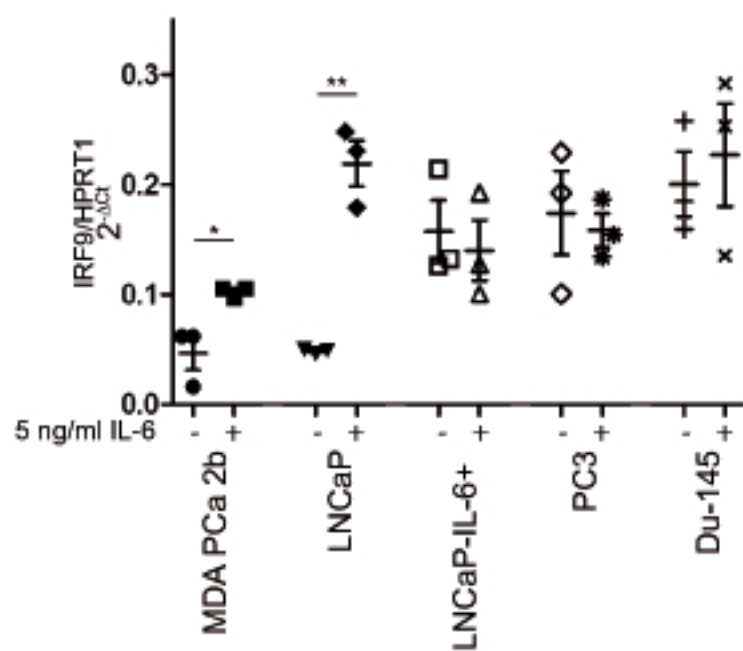**B**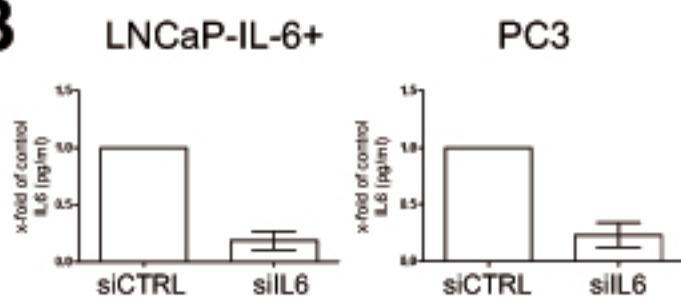

Fig.S4 Erb et al., 2013

Supplement: Supplementary data [file supp_ERC-13-0222_Supplementary_figure_4.pdf]
